# How Accurate Are Accuracy-Nudge Interventions? A Preregistered Direct Replication of Pennycook et al. (2020)

**DOI:** 10.1177/09567976211024535

**Published:** 2021-06-11

**Authors:** Jon Roozenbeek, Alexandra L. J. Freeman, Sander van der Linden

**Affiliations:** 1 Department of Psychology, University of Cambridge; 2 Winton Centre for Risk and Evidence Communication, University of Cambridge

**Keywords:** social media, misinformation, fake news, accuracy nudge, priming, open data, open materials, preregistered

## Abstract

As part of the Systematizing Confidence in Open Research and Evidence (SCORE) program, the present study consisted of a two-stage replication test of a central finding by Pennycook et al. (2020), namely that asking people to think about the accuracy of a single headline improves “truth discernment” of intentions to share news headlines about COVID-19. The first stage of the replication test (*n* = 701) was unsuccessful (*p* = .67). After collecting a second round of data (additional *n* = 882, pooled *N* = 1,583), we found a small but significant interaction between treatment condition and truth discernment (uncorrected *p* = .017; treatment: *d* = 0.14, control: *d* = 0.10). As in the target study, perceived headline accuracy correlated with treatment impact, so that treatment-group participants were less willing to share headlines that were perceived as less accurate. We discuss potential explanations for these findings and an unreported change in the hypothesis (but not the analysis plan) from the preregistration in the original study.

The spread of online misinformation and fake news has long been considered a threat to science and society (Lewandowsky et al., 2017; Lewandowsky & van der Linden, 2021), but it has taken on renewed urgency during the novel coronavirus SARS-CoV-2 (COVID-19) pandemic. For example, belief in misinformation about the virus has been linked to violent intentions (Jolley & Paterson, 2020), reduced self-reported willingness to comply with health-guidance measures (such as mask wearing), and lower willingness to get vaccinated against the disease (Loomba et al., 2021; Roozenbeek et al., 2020). The wide proliferation of COVID-19-related misinformation, which includes claims that the SARS-CoV-2 virus was manufactured in a military laboratory in Wuhan, China, and that 5G radiation worsens coronavirus symptoms (World Health Organization, 2021), has prompted the World Health Organization to warn of an ongoing “infodemic” (Zarocostas, 2020).

In light of the onslaught of fake news, the demand for evidence-based interventions that could help reduce the spread of misinformation is high, but supply remains relatively low (Van Bavel et al., 2020; van der Linden & Roozenbeek, 2020)—with some notable exceptions (Basol et al., 2021; Guess et al., 2020; Maertens et al., 2021). Recent work has examined how simple interventions that prime (or “nudge”) people to think about the accuracy of the information that they see online can reduce the spread of misinformation (Pennycook et al., 2021). Fazio (2020), for example, showed that pausing to consider why a headline is true or false contributes to lower self-reported willingness to share false news.

The most notable example of such an accuracy-nudge intervention in the context of COVID-19 misinformation is the subject of this replication. In a recent study, Pennycook et al. (2020) found that “a simple accuracy reminder at the beginning of the study . . . nearly tripled the level of truth discernment in participants’ subsequent sharing intentions” (p. 770). In their study, which was recently replicated in three preregistered experiments in the context of U.S. political misinformation by Pennycook et al. (2021), each participant was randomly assigned to a treatment or a control group. Treatment-group participants were shown a single headline unrelated to COVID-19 and were asked the following question: “To the best of your knowledge, is the above headline accurate?” (yes/no response options). After this accuracy nudge, both the treatment and control group were shown 15 real and 15 false headlines related to COVID-19 and asked the following question for each headline: “If you were to see the above on social media, how likely would you be to share it?” (response options ranged from *extremely unlikely* to *extremely likely* on a 6-point scale). The authors found that, compared with the control group (*d* = 0.05), the treatment group (*d* = 0.14) displayed significantly higher truth discernment, which the authors defined as “the extent to which individuals distinguish between true and false content in their judgments” (p. 772).

This finding garnered significant press attention and was reported as one that could “curtail COVID-19 misinformation online” (Walsh, 2020). The findings of this study have high theoretical relevance in that the authors are advancing a largely cognitive-inattention-based account of misinformation sharing that directly challenges a motivational account of why people share fake news (Pennycook et al., 2021; Pennycook & Rand, 2019). In other words, the authors argue that people tend to share false information about COVID-19 on social media more because they fail to think about accuracy rather than because they have an a priori motivation to share content that is consistent with their beliefs (Van Bavel & Pereira, 2018). On an applied level, these results are also relevant because a direct consequence of the inattention-based account is that sharing behavior is amenable to accuracy interventions. In fact, as the authors recommend in their article, accuracy nudges are tools “that social media platforms could directly implement” (Pennycook et al., 2020, p. 778). However, as IJzerman et al. (2020) note, caution is advised when applying behavioral-science research directly to policy (particularly during the COVID-19 pandemic), as the evidence readiness of research findings is not always clear. Replicating high-profile findings to assess the quality of the available evidence that informs policies directed at combating the spread of misinformation is therefore of the utmost importance. In light of this, we were invited to replicate the key findings reported in Study 2 by Pennycook et al. (2020; referred to throughout this article as the “target study”) as part of a large-scale replication project conducted by the Center for Open Science (COS)’s Systematizing Confidence in Open Research and Evidence (SCORE) program (https://www.cos.io/score).

**Statement of Relevance**

Misinformation about COVID-19 is a significant societal challenge. A recent high-profile intervention by Pennycook et al. (2020) found that subtly priming people to consider the accuracy of news headlines improves the quality of people’s intentions to share news about COVID-19 on social media. This high-powered, preregistered replication of Pennycook et al.’s study offers nuance to the existing body of research on accuracy nudges. In the first stage of data collection, we found no effect of the accuracy nudge on subsequent sharing intentions. After collecting a second round of data with additional participants, we did find a significant treatment effect in the combined data set. Truth discernment in the treatment group improved at about 1.4 times the rate that it did in the control group. This represents an intervention effect around half (50%) of that reported in the original study. We encourage further research into the effectiveness of accuracy nudges across domains, including potential moderators and whether their effects decay over time.

## Method

The preregistration for this replication project was coordinated and verified according to COS protocols as part of the SCORE program and can be found on OSF (https://osf.io/rkfq5/). The focal hypothesis that we replicated in this study was determined through SCORE and stemmed from Study 2 of Pennycook et al. (2020): There is a significant positive interaction between headline veracity (true or false headline) and treatment (accuracy induction) predicting likelihood to share. Specifically, the treatment condition should increase participants’ discernment. Criteria for a successful replication attempt was finding a statistically significant effect (α = .05, two tailed) in the same pattern as was found in the target study on the focal-hypothesis test.

Pennycook et al. (2020) used truth discernment as their outcome measure. However, in their preregistration for Study 2, the authors hypothesized that the accuracy nudge would “decrease the likelihood that [participants] will be willing to share *false* [emphasis added] information about COVID-19 on social media.”^1^ Importantly, the truth-discernment finding reported in the published article was actually driven by a different pattern of headline ratings: There was an increase in sharing *real* news headlines (as opposed to a reduction in sharing false headlines). As reported, the intervention did not reduce sharing of false headlines, “sharing intentions for true headlines were significantly higher than for false headlines, *d* = 0.142, 95% CI = [0.049, 0.235]” (Pennycook et al., 2020, p. 776).^2^ To remedy this ambiguity and further unpack “truth discernment,” we therefore preregistered two directional hypotheses. Hypothesis 1 was that prompting people to think about accuracy decreases the likelihood that they will be willing to share false information about COVID-19 on social media. Hypothesis 2 was that prompting people to think about accuracy increases the likelihood that they will be willing to share true information about COVID-19 on social media.

### Sample

The replication procedure used for the SCORE program followed the same approach used by Camerer et al. (2018). Power calculations were done in accordance with the guidelines of the Social Sciences Replication Project (SSRP), which states that the first round of data collection must achieve 90% power to detect 75% of the original effect size. In our case, this meant we recruited 701 participants for a total of 21,030 headline ratings. The SCORE guidelines state that a second round of data must be collected only if the replication is unsuccessful after the first round. The pooled sample (both the first and second stage) must achieve 90% power to detect 50% of the original effect size; in our case, this meant that an additional 882 participants (or 26,443 ratings) would be recruited if the replication failed in the first round, for a total of 1,583 participants in the pooled sample. The full preregistered power analysis for this replication can be found on OSF at https://osf.io/rkfq5/.

The target study by Pennycook et al. (2020) used a sample that was “matched to the U.S. population on age, gender, ethnicity, and [geographic] region” but was “not obtained via probability sampling and therefore should not be considered truly nationally representative” (p. 778). For this replication, we also opted to collect national quota samples that were matched to the U.S. population on age, gender, ethnic background, and geographic region but from a different platform, namely Respondi, a panel provider that is certified by the International Organization for Standardization (respondi.com). Respondi relies on an actively managed online access panel using both off-line and online recruitment strategies. Participants receive credit for participation in online surveys, and the U.S. panel is composed of about 120,000 participants. A random sample is drawn from the panel, which is then stratified and matched on national quotas. The full quota for each group can be found in Table S1 in the Supplemental Material. The data, analysis and visualization scripts, and STATA output for this study can be found on OSF at https://osf.io/rkfq5/.

### Procedure

To ensure that our study would be an honest and fair attempt to replicate the original findings, independent reviewers identified by COS as part of the SCORE program evaluated our detailed preregistration. Although we followed the original study’s protocol as closely as possible (the authors uploaded their Qualtrics surveys and data-analysis scripts to their OSF page, and we made only minor adaptations), we deviated from the target study in one important way: Some of the true and false headlines about COVID-19 that were used originally were outdated when we conducted our replication. We therefore selected a series of new headlines to use in our study, which were kindly provided and approved by the original authors. Participants were paid 1 British pound sterling for completing the survey. Ethical approval for this study was granted by the Cambridge Psychology Research Ethics Committee (PRE.2020.086) and the U.S. Army Human Research Protection Office (HR00112020015).

### Measures

#### Headline-rating task

As in the target study, participants in both the treatment and control groups were shown 15 real and 15 false headlines related to COVID-19 in a random order. These headlines were designed to mimic the format of Facebook posts, including an image, a headline, and a lede sentence. For each headline, participants were asked the following question: “If you were to see the above on social media, how likely would you be to share it?” Possible answers ranged from *extremely unlikely* to *extremely likely* on a 6-point scale. Before starting the task, treatment-group participants rated the accuracy of a single headline that was unrelated to COVID-19. Following the target study, we framed this as being part of a pretest. Each participant was randomly shown one of four possible headlines, which were kept the same as in the target study.

#### Additional questions

Aside from the headline-rating task, participants in both the treatment and control groups answered another series of questions. Before the headline-rating task, participants were asked what type of social media accounts they use. Following the target study and our preregistration, we excluded them from the rest of the study if they indicated using neither Facebook nor Twitter. Next (also before the headline-rating task), participants were asked two questions about the COVID-19 pandemic. The first was, “How concerned are you about COVID-19 (the new coronavirus)?” which they answered on a sliding scale from 0 (*not concerned at all*) to 100 (*extremely concerned*). In addition, they were asked, “How often do you proactively check the news regarding COVID-19 (the new coronavirus)?” which they answered on a scale from 1 (*never*) to 5 (*very often*).

After the headline-rating task, and again following the target study, participants were shown a reworded six-item cognitive reflection test (CRT; see Frederick, 2005; Thomson & Oppenheimer, 2016), which is associated with reflective thinking; a general science-knowledge quiz (as a measure of background knowledge about issues related to science), consisting of 17 questions about basic science facts such as “antibiotics kill viruses as well as bacteria” (McPhetres et al., 2019); and the Medical Maximizer-Minimizer Scale (MMS), which measures to what extent people are “medical maximizers” who seek medical treatment for minor health issues or “medical minimizers” who are more likely to avoid seeking treatment unless absolutely necessary (Scherer et al., 2016). Finally, participants were asked about their political ideology in terms of social and economic issues, as well as their party alignment: whom they voted for in the 2016 U.S. presidential elections, party identification (Democrat/Republican/independent/other), and party identification on a 6-point scale ranging from *strongly Democrat* to *strongly Republican*.

#### Attention checks

Following the target study, we included three attention checks in the form of subtle instructions in the middle of question blocks (Berinsky et al., 2014). Following Pennycook et al. (2020) and our preregistration, we did not exclude participants who failed attention checks from analysis, but we report results for different levels of attentiveness.

### Analyses

We use two main methods of analysis to test our focal hypothesis. First, following the target study, we conducted an analysis at the level of the rating using linear regression with robust standard errors clustered on participants and headlines. To do so, we followed the analysis script (written in STATA) that the original authors posted on OSF.^3^ Sharing intentions on the 6-point Likert scale were rescaled so that 1 was 0 and that 6 was 1 (as in the target study). Our outcome variable of interest was the interaction between treatment condition and headline veracity (i.e., sharing intentions for real headlines minus false headlines, which we call *discernment*), which shows to what extent discernment differs between the treatment and control condition. Second, to test Hypotheses 1 and 2, we performed standard independent-samples *t* tests and Bayesian *t* tests at the participant level to determine whether there was a significant difference between the treatment and control conditions for false and real headlines as well as discernment.

## Results

### Stage 1

In the first stage of data collection, we recruited 701 participants (51.4% female; age: *M* = 45.6, *SD =* 16.2; 75.0% White/Caucasian; see Table S2 in the Supplemental Material for a full overview of the sample composition). Following the original authors’ analysis and our preregistration, we conducted a linear regression with robust standard errors clustered on participants and headline (using STATA’s *ivreg2* package; Baum et al., 2003). This analysis yielded no significant interaction between headline veracity and treatment, β = 0.0046, 95% confidence interval (CI) = [−0.016, 0.026], *F*(3, 21030) = 1.53, *p* = .67 (see Table S5 in the Supplemental Material).^4^ In addition, to ensure this was not due to minor differences in the clustering algorithm, we ran the same analysis using STATA’s *reghdfe* package (which uses high-dimensional fixed-effects linear regression; Correia, 2016), and these also yielded no significant interaction between headline veracity and treatment, β = 0.0046, 95% CI = [−0.018, 0.027], *F*(3, 21030) = 1.53, *p* = .68 (see also Table S5). Thus, according to the criteria used for the SCORE program, the replication was unsuccessful in the first stage.

### Stage 2

For Stage 2, we recruited an additional 882 participants, for a total pooled sample of 1,583 participants (51.4% female; age: *M* = 45.4, *SD* = 16.3; 75.0% White/Caucasian; see Table S2). Following our preregistration, we estimated the interaction term using only one package, *ivreg2*, as the replication method for SCORE purposes. This was done to prevent the possibility of incongruence of results between methods for the pooled sample and to bring this analysis plan in line with other SCORE replications, which just use one statistical test as evidence for the macro project. We did not preregister a correction for the two-stage data-collection process, so we present results here for the pooled sample according to our preregistration (i.e., without applying a correction to control the family-wise error rate).

**Fig. 1.**
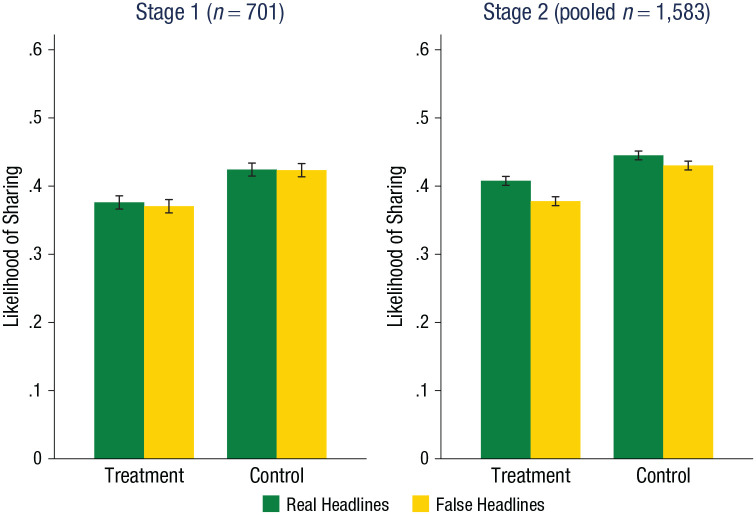
Bar graph showing the *z*-scored likelihood of sharing real and false headlines in the treatment and control conditions, separately for Stage 1 data and Stage 2 data pooled after further data collection. Error bars represent 95% confidence intervals.

This analysis yielded a significant interaction effect between headline veracity and treatment, β = 0.015, 95% CI = [0.0027, 0.027], *F*(3, 47490) = 4.52, *p* = .017; treatment-group effect size: *d* = −0.14, 95% CI = [−0.17, −0.12] (see Table S6 in the Supplemental Material). The effect size for the control group was directionally similar (*d* = −0.10, 95% CI = [−0.13, −0.078]), but sharing discernment was still 1.4 times higher in the treatment group than in the control group, an attenuation of about 50% compared with the effect sizes reported in the target study. Figure 1 shows the results in a bar graph.

Following our preregistration, we performed several additional analyses. First, we replicated the main analysis using high-dimensional fixed-effects linear regression (with STATA’s *reghdfe* package), as we did in Stage 1 of data collection. Doing so also yielded a significant (uncorrected) effect for the interaction of headline veracity and treatment, β = 0.015, 95% CI = [0.0020, 0.028], *F*(3, 47490) = 4.52, *p* = .026 (see Table S6).

Second, we conducted independent-samples and Bayesian *t* tests to evaluate differences in sharing intentions between the treatment and control groups for discernment, as well as for each individual headline (Hypotheses 1 and 2). Doing so yielded a significant (noncorrected) effect for discernment (treatment: *M* = 0.26, control: *M* = 0.19, mean difference: −0.075, 95% CI = [−0.15, −0.0019]), *t*(1581) = −2.013, *p* = .044, *d =* −0.10, 95% CI = [−0.20, −0.0025]. A Bayesian *t* test revealed a Bayes factor (BF) indicating that the data are approximately 1.7 times more likely to occur under the focal hypothesis than under the null hypothesis (BF_10_ = 1.705, *M* = −0.061, 95% CI = [−0.17, 0.016], error percentage = 7.148 × 10^−6^), which is weak (or anecdotal) evidence in support of the focal hypothesis and against the null hypothesis that there would be no difference in truth discernment between the treatment and control groups (van Doorn et al., 2021; see Table S4 in the Supplemental Material).^5^

We found that—unlike in the target study—an independent-samples *t* test showed a significant difference for average willingness to share false headlines, in the sense that the treatment group was significantly less willing to share false headlines than the control group (treatment: *M* = 2.89, control: *M* = 3.04, mean difference: *=* 0.15, 95% CI = [0.011, 0.29]), *t*(1581) = 2.113, *p* = .035, *d =* 0.11, 95% CI = [0.0075, 0.21]. Similarly, a Bayesian *t* test gave weak support in favor of Hypothesis 1 (BF_10_ = 1.948, *M* = 0.066, 95% CI = [−0.013, 0.170], error percentage = 0.0030). Also unlike the target study and against our Hypothesis 2, our analyses found no significant difference for real headlines (treatment: *M* = 3.15, control: *M* = 3.23, mean difference: 0.075, 95% CI = [−0.057, 0.21]), *t*(1581) = 1.110, *p* = .27, *d =* 0.06, 95% CI = [−0.043, 0.15]. A Bayesian *t* test provided weak support against Hypothesis 2; treatment-group participants indicated being *less* willing to share real news headlines than control-group participants (BF_10_ = 0.732, *M* = 0.028, 95% CI = [−0.040, 0.12], error percentage = 5.15 × 10^−7^; see Tables S3 and S4 in the Supplemental Material).

Third, previous research has generally found that priming effects dissipate rapidly, within a span of seconds (Branigan et al., 1999; Trammell & Valdes, 1992), and such decay would have important implications for the practical translation of accuracy primes. Following our preregistration, we therefore conducted an exploratory analysis to examine whether the accuracy-nudge effect was distributed more or less equally across the duration of the headline-rating task or whether it was predominantly observed immediately after the accuracy induction. To do so, we recorded the display order in which each of the 30 headlines were shown to each participant. This was a slight deviation from our preregistration, as Qualtrics did not allow us to export the time elapsed between the start of the rating task and the moment a headline was shown to the participant. We therefore considered looking at the display order of headlines (which were shown in a random order) to be a useful proxy for the time between being exposed to the accuracy nudge and rating an individual headline. A linear regression showed that the interaction among treatment, condition, and headline order (1–30) was marginal, β = −0.00066, 95% CI = [−0.013, −0.00043], *F*(7, 47490) = 4.49, *p* = .052. This result suggests that there is no strong evidence for linear decay of the accuracy-nudge effect but does not rule out nonlinear decay, particularly the possibility that the treatment effect conferred by the accuracy nudge occurs disproportionately for the first few headlines and tapers off thereafter. We examine this possibility in Analysis S1 in the Supplemental Material.

Fourth, following Pennycook et al. (2020), we replicated the main analysis for different levels of attentiveness (defined as the number of attention checks successfully passed by the participants, with a maximum of three). Unlike the target study, our study found that the accuracy nudge was no longer significant for participants who passed two or more attention checks, β = 0.0135, 95% CI = [−0.00084, 0.028], *F*(3, 36930) = 3.66, *p* = .065; this was also the case for attentive participants who passed all three attention checks, β = 0.0065, 95% CI = [−0.021, 0.033], *F*(3, 13290) = 3.87, *p* = .64 (see Table S7 in the Supplemental Material). However, we found no significant three-way interaction among condition, sharing discernment, and attentiveness, β = −0.029, 95% CI = [−0.088, 0.030], *F*(7, 47490) = 17.91, *p* = .337.

Fifth, we checked whether the accuracy nudge was effective across the political spectrum. We found a significant effect of treatment on sharing discernment for Democrats, β = 0.0195, 95% CI = [0.0037, 0.035], *F*(3, 32910) = 6.74, *p* = .016, but not for Republicans, β = 0.00764, 95% CI = [−0.0073, 0.023], *F*(3, 14580) = 0.36, *p* = .315. Furthermore, we found that the nudge was effective for participants who did not vote for Donald Trump in the 2016 elections, β = 0.0231, 95% CI = [0.079, 0.039], *F*(3, 32490) = 6.58, *p* = .003, but not for participants who did vote for Trump, β = 0.00376, 95% CI = [−0.013, 0.020], *F*(3, 15000) = 0.12, *p* = .653. However, the three-way interactions among condition, sharing discernment, and identifying as Republican (*p =* .283) and voting for Trump (*p* = .082) were not significant.

Sixth, we checked for three-way interactions among condition, sharing discernment, and (a) performance on the CRT, (b) scientific knowledge, and (c) scores on the MMS. We found (unlike the target study) a significant three-way interaction with MMS scores (*p* < 0.001) but not with partisanship, CRT, or scientific knowledge (consistent with the target study; see Table S8 in the Supplemental Material).

**Fig. 2.**
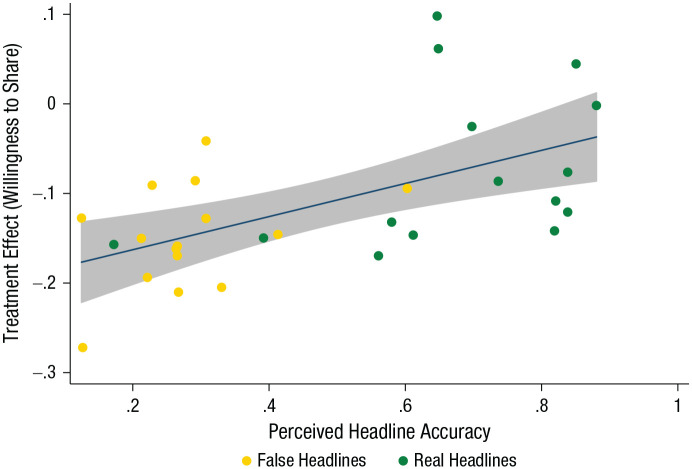
Scatterplot (with best-fitting regression line) showing the association between perceived headline accuracy (from a separate data set that replicated the accuracy condition in the target article’s Study 1) and the effect of the treatment (accuracy nudge) on willingness to share real and false headlines. The error band represents the 95% confidence interval.

Finally, as in the target study, we checked whether perceived headline accuracy (from the original article’s Study 1, in which participants were asked to indicate the accuracy of all 30 headlines) was correlated with the impact of the accuracy nudge on sharing discernment.^6^ As in the target study, we found that perceived headline accuracy was significantly correlated with treatment impact (*r* = .57, *p* = .001): Treatment-group participants indicated being less willing to share headlines that were also generally perceived as less accurate. This is in line with the mechanism proposed by Pennycook et al. (2020) for the accuracy-nudge effect. These results are shown in Figure 2 (see also Table S9 in the Supplemental Material).

## Discussion and Conclusion

The study by Pennycook et al. (2020) that was the subject of this replication garnered significant media attention at the time of its publication because of its promise of a potential intervention to protect against the damaging spread of fake news about COVID-19. In our independent replication, we found that the first stage of data collection, calculated to achieve 90% power to detect 75% of the target study’s effect size, was unsuccessful: We found no significant difference between the treatment and control groups in truth discernment (*p* = .67). After a second stage of data collection (the pooled sample size was powered at 90% to detect 50% of the original effect size), we replicated the treatment effect. Yet whereas truth discernment was about 2.8 times higher in the treatment group (relative to the control group) in the original study (treatment: *d* = 0.14, control: *d* = 0.05), there was about 50% attenuation in our second-stage replication, so the treatment effect was just 1.4 times higher (treatment: *d* = 0.14, control: *d* = 0.10). This difference appears to have been driven by higher baseline discernment in the control group. We did find that the mechanism proposed by the target authors—namely that the treatment impact on sharing intentions conferred by the accuracy nudge should correlate with the perceived accuracy of news headlines—was present in our replication. Our findings are thus in partial agreement with those of the target study, as well as with a recent study by the same authors that found significant effects of accuracy nudges on sharing intentions in three separate experiments, including a Twitter study (Pennycook et al., 2021).

We offer several interpretations for why the first stage of our replication was unsuccessful and why the intervention effect size was lower than those reported in the target and other similar studies. First, because our results pertain to COVID-19 misinformation and our replication was conducted at a later stage in the COVID-19 pandemic than the target study, people may have become more attuned to COVID-19 misinformation over time, which may have resulted in a reduced treatment impact. In other words, it is possible that the effectiveness of accuracy nudges varies across time and issue domains.

Second, we used a different headline set than the target study, as some of the original headlines were outdated by the time we conducted our replication. It is possible that item effects (i.e., individual headlines having a disproportionate impact on the overall results) impacted our findings. However, we note that we changed only a few headlines and that the majority of the target study’s headlines were retained—the remainder were selected and provided by the target authors themselves to maximize replication potential.

Third, the target study was conducted on the participant-recruitment platform Lucid, and other studies on accuracy nudges (Pennycook et al., 2021) were conducted on Amazon’s Mechanical Turk, whereas we used Respondi in our replication to recruit participants. Lucid and Mechanical Turk are known to have high levels of inattentive responding (Aguinis et al., 2021; Aronow et al., 2020; Barends & de Vries, 2019). In our replication, we found that attentive participants (i.e., participants who passed all three attention checks) were less responsive to the accuracy nudge than inattentive participants (although we note that this effect was not observed in the target study, and in our replication, a three-way interaction among treatment, discernment, and attentiveness was not significant). We therefore encourage further research to untangle whether participant inattentiveness during online surveys (in contrast with general inattentiveness) has an influence on the effectiveness of accuracy nudges.

Fourth, previous research has found that priming effects are subject to decay in a matter of several seconds (Branigan et al., 1999; Trammell & Valdes, 1992). Our replication was the first to explore the decay of the accuracy-nudge effect over time. We found some support for the idea that the nudge effect occurs predominantly for the first several headlines that are shown, although we note that this analysis is merely exploratory (see Analysis S1 for a discussion). We encourage further research into the decay of accuracy nudges and other fake-news interventions (Basol et al., 2021; Maertens et al., 2021).

Finally, we discuss a discrepancy between the target study’s preregistered hypothesis and the reported results. The preregistration contains the following question: “does prompting people to think about accuracy decrease the likelihood that they will be willing to share false information about COVID-19 on social media?” It further states that “the treatment effect is predicted to be larger for fake news.” However, Pennycook et al. (2020) did not find that participants who were exposed to the accuracy nudge were less willing to share false headlines: “although participants in the control condition were not significantly more likely to say that they would share true headlines compared with false headlines . . . in the treatment condition, sharing intentions for true headlines were significantly higher than for false headlines” (p. 776). Although the authors’ preregistered analysis plan (which focused on sharing discernment) is consistent with the reported analysis, the preregistration’s main hypothesis is not mentioned in the published article. Truth discernment can improve for a variety of reasons, and it is therefore important to be clear about the possible theoretical basis behind why an intervention is expected to improve truth discernment, either by acting on fake-news sharing, real-news sharing, or both. It is important to note that the pattern reported by Pennycook et al. (2020), namely, via a difference in sharing of real headlines, is different from the hypothesis originally preregistered by the authors (i.e., via a difference in sharing of false headlines). Their hypothesis actually matches the pattern reported in this replication, in which we found that treatment-group participants were less willing to share false headlines (*p* = .035, *d* = 0.11), but we found no difference for real headlines (*p* = .27, *d* = 0.06; see Table S4).

If we include the three studies from Pennycook et al. (2021), which used highly similar study designs, accuracy-nudge studies have in fact so far yielded three different patterns: an increased self-reported willingness to share real headlines for treatment participants (in the target study), no difference between the treatment and control groups (Stage 1 of our replication), and a decreased self-reported willingness to share false headlines (Stage 2 of our replication and Studies 3, 4, and 5 in Pennycook et al., 2021). On balance, separate studies seem to show that accuracy nudges predominantly affect people’s intentions to share false headlines. In addition, in support of the authors’ own posited mechanism, results showed that the perceived accuracy of the headlines correlated with the treatment effect across different studies.

It could be argued that whether the accuracy nudge affects intentions to share real or false headlines is both theoretically and practically irrelevant in the context of social media, as people’s feeds can contain only a small chunk of all existing misinformation, and increasing the ratio of real news versus false news (i.e., improving the quality of people’s sharing decisions) reduces the amount of fake news that social media users are potentially exposed to (Pennycook et al., 2021). However, a counterargument to this is that it is not only the amount of misinformation on people’s social media feed that matters. The type of misinformation matters, too. It is difficult to predict how harmful a given piece of misinformation is going to be, and individual viral news stories can cause significant harm (van der Linden & Roozenbeek, 2020). Furthermore, accuracy nudges aim to reduce the likelihood that people share misinformation with others but do not contain any training to reduce people’s susceptibility to misinformation. Interventions aimed at reducing susceptibility to misinformation specifically may therefore also be necessary, and hence the specific pattern underlying truth discernment remains relevant for effectively targeting the detection of real versus fake news. We therefore call for further theorization into the mechanisms behind how—and circumstances under which—accuracy nudges impact subsequent news-sharing intentions.

Jointly, our findings have important implications for future research on the social and cognitive science of misinformation and simple interventions to combat the spread of fake news. First, the effect size of the accuracy-nudge intervention appears small, and we advise scholars that variation can be expected across replications. Second, clearer a priori theorizing is needed to explain how interventions are expected to impact real-news detection, fake-news detection, and veracity discernment. Third, researchers seeking to investigate the effectiveness of accuracy nudges in future studies should explore (nonlinear) decay over time using longitudinal designs, consider potential moderators such as partisanship and trust in media, and consider to what extent the effect size of accuracy nudges is domain or issue dependent.

## Supplemental Material

sj-docx-1-pss-10.1177_09567976211024535 – Supplemental material for How Accurate Are Accuracy-Nudge Interventions? A Preregistered Direct Replication of Pennycook et al. (2020) Click here for additional data file. Supplemental material, sj-docx-1-pss-10.1177_09567976211024535 for How Accurate Are Accuracy-Nudge Interventions? A Preregistered Direct Replication of Pennycook et al. (2020) by Jon Roozenbeek, Alexandra L. J. Freeman and Sander van der Linden in Psychological Science

## Transparency

*Action Editor:* Patricia J. Bauer

*Editor:* Patricia J. Bauer

*Author Contributions*

A. L. J. Freeman initiated the Systematizing Confidence in Open Research and Evidence (SCORE) project. J. Roozenbeek programmed the experiment and collected and analyzed the data under the supervision of S. van der Linden and A. L. J. Freeman. J. Roozenbeek and S. van der Linden drafted the manuscript, and A. L. J. Freeman provided feedback. All authors approved the final manuscript for submission.

**Open Practices:** All data, materials, and preregistration information for the current study have been made publicly available via OSF and can be accessed at https://osf.io/rkfq5/. This article has received the badges for Open Data, Open Materials, and Preregistration. More information about the Open Practices badges can be found at http://www.psychologicalscience.org/publications/badges.

## Notes

1. The target authors’ preregistration can be found here: https://osf.io/2jtx6/.

2. We note that the effect size reported in the target study is for the treatment group only. The effect size for the control group was as follows: *d* = 0.05, 95% CI = [0.016, 0.085]), which means that “sharing discernment (the difference in sharing likelihood of true relative to false headlines) was 2.8 times higher in the treatment condition compared with the control condition” (Pennycook et al., 2020, p. 776).

3. With made one minor change: Instead of the external *cluster2* STATA package, we relied on the more commonly used, built-in *ivreg2* and *reghdfe* STATA packages to estimate the linear regression, which both reproduced the same results as those reported by Pennycook et al. (2020) when performed on the original data set.

4. The full output of the STATA regression models and all analyses conducted in this study, along with the code itself, can be found on OSF at https://osf.io/rkfq5/.

5. The prior we used is described by a Cauchy distribution centered around 0 and with a width parameter of 0.05, corresponding to a 79% chance that the effect size lies between −0.15 and 0.15, in line with the effect sizes reported in the target study by Pennycook et al. (2020).

6. To do so, following our preregistration, we partially replicated Study 1 from the target article. Specifically, we replicated that study’s accuracy condition, in which participants were asked to indicate the accuracy of the same 30 headlines used in this study (yes/no).
