# Supplementary material for: How Accurate Are Accuracy-Nudge Interventions? A Preregistered Direct Replication of Pennycook et al. (2020)
Source: Psychol Sci. 2021 Jun 11;32(7):1169–78. doi: 10.1177/09567976211024535 (PMC8641132; doi:10.1177/09567976211024535)
Supplement: sj-docx-1-pss-10.1177_09567976211024535 – Supplemental material for How Accurate Are Accuracy-Nudge Interventions? A Preregistered Direct Replication of Pennycook et al. (2020) [file sj-docx-1-pss-10.1177_09567976211024535.docx]

**Supplementary Information for**

**How accurate are accuracy nudge interventions? A pre-registered direct replication of Pennycook et al. (2020).**

**Deviations from the pre-registration**

First, it turned out not to be possible to record the time elapsed between the start of the sharing task and the moment participants were shown a particular headline. Instead, we therefore exported the viewing order of the randomised headlines, using Qualtrics’ “Export viewing order data for randomized surveys” function. Second, we were unable to control for whether the treatment interacts with the nearest distance to the epicentre of a COVID-19 outbreak. Third, we conducted our analyses in STATA instead of R, following the original paper’s analysis and the STATA code published on the OSF by the original authors. Fourth, due to an error in the survey implementation, we were unable to include the pre-registered numeracy measures and rational versus intuitive style decision-making as correlates. Fifth, in the second stage of data collection, we recruited one more participant than necessary (1,583 versus the pre-registered 1,582).

**Supplementary Analysis S1: Decay of the accuracy nudge effect over time**

Previous research on priming has shown that priming effects have a tendency to dissipate rapidly, i.e., within a span of several seconds (Branigan et al., 1999; Trammell & Valdes, 1992). Within the context of accuracy nudges, exploring to what extent their impact on people’s sharing decisions is subject to such decay is a key question with important implications for the implementation of accuracy nudges in online environments, of which we offer a first exploration here. As we did not offer any explicit hypotheses or analysis plans for the decay function in our pre-registration, we consider the analysis below to be exploratory and post-hoc, but our descriptive findings may be used as a guide for future research.

As mentioned in the main text of this study, we use headline display order as a proxy for the time elapsed between the start of the headline rating task and the moment a particular headline was shown to a participant. Headline display order was randomized (i.e., the 30 headlines that participants were asked to rate on a 1-6 sharing intentions scale were shown in a random order), so if for participant A headline 1 was the third out of 30 headlines shown, headline display order for headline 1 would have the value 3 for this participant, and so on. As reported in the main text, we did not find strong evidence that the nudging effect decays linearly over time, i.e., the interaction between treatment condition, discernment and headline display order was marginally significant: *β* = -0.0.0066, 95%CI [-0.013, -0.00043], *F*(7,47490) = 4.49, *p* = 0.052.

However, the absence of a significant *linear* interaction effect does not mean that decay is not occurring; after all, if priming decay generally occurs after several seconds, it is conceivable that the accuracy nudge effect is predominantly observed in the first few headlines that participants are asked to rate. In other words, a linear decay curve may not be the best fit for accuracy nudge decay. To explore these questions, we looked at participants’ sharing intentions of true and false headlines, as well as sharing discernment, at each headline display order (1-30). The results are shown in Figure S1.


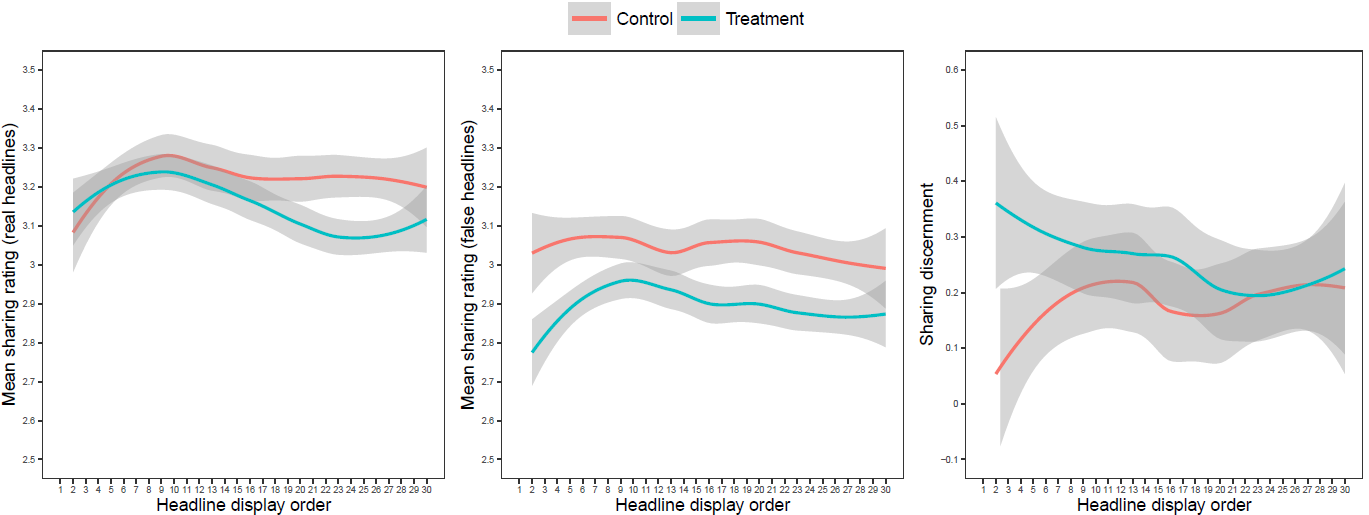


*Figure S1*. Mean ratings for “willingness to share” for real headlines (left), false headlines (middle) and sharing discernment (right) by headline display order for the pooled sample (*N* = 1,583).

Descriptively, Figure S1 shows that mean sharing ratings for the real headlines are roughly the same for the treatment and control group, although treatment group participants appear to indicate sharing real headlines *less* than the control group between display order 20 and 29. Conversely, for false headlines, sharing intentions mostly resemble a flat line for the control group, but are lower for the treatment group, especially for approximately the first 7 headlines (less so afterwards). Accordingly, the right graph in Figure S1 shows that the difference in sharing discernment between the treatment and control groups is therefore largest for the first few headlines.

To double-check this exploratory finding, we plotted the treatment x discernment coefficient (i.e., the impact of the accuracy nudge on sharing discernment) against the headline display order. The results are shown in Figure S2 for both the pooled sample (*N =* 1,583) and the second round of data collection (*n* = 882). The patterns are similar to those shown in Figure S1: descriptively, the treatment x discernment coefficient is clearly positive for the first 7 headlines, but randomly positive or negative for the remaining 23 headlines.

| 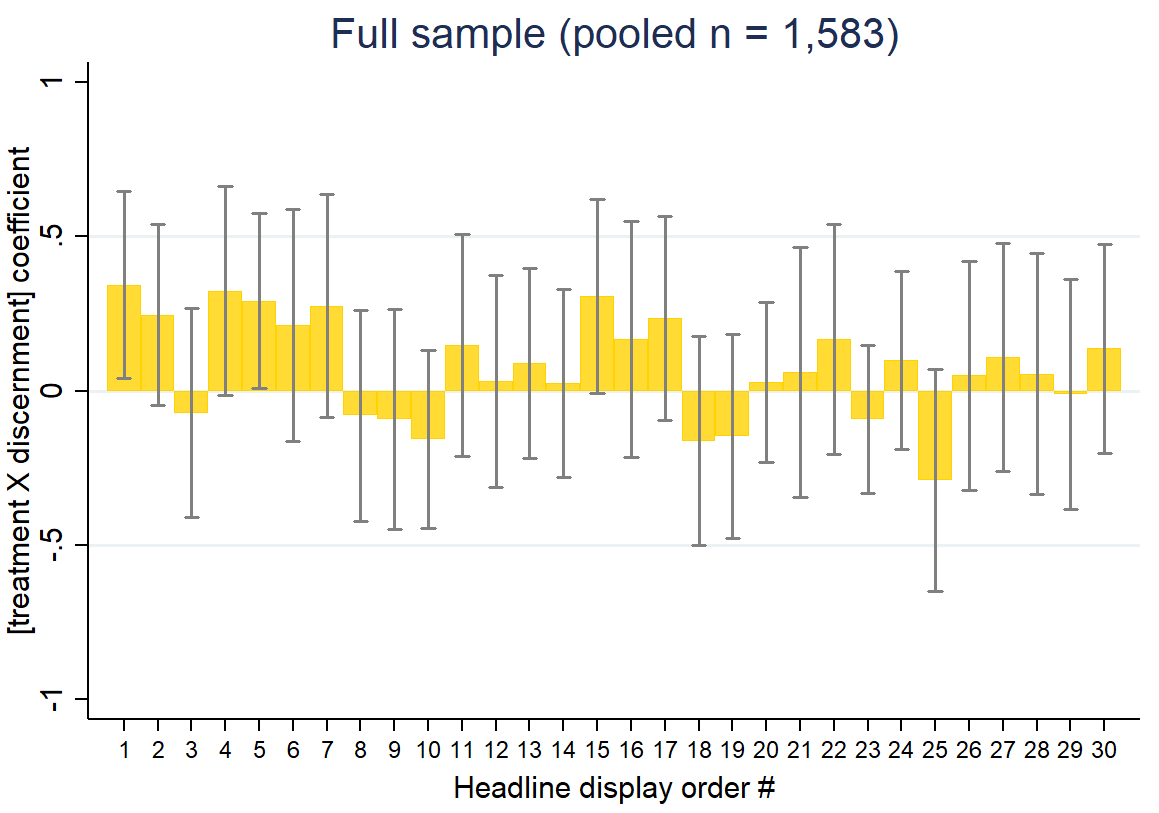 | 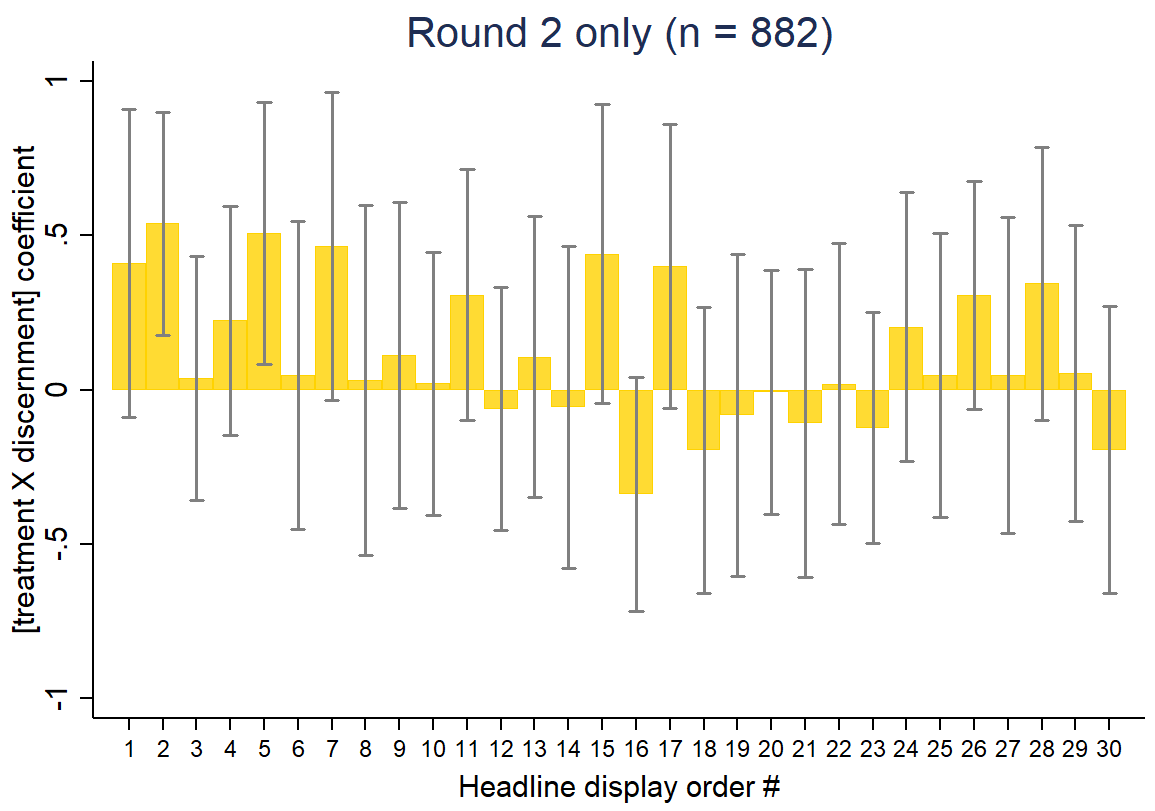 |
| --- | --- |

*Figure S2*. Bar graph of treatment x discernment coefficient by individual headline order, for the full sample and round 2 of data collection. A larger coefficient represents a larger effect of the accuracy nudge on sharing discernment. Error bars represent standard errors.

Accordingly, just for illustrative and exploratory purposes, we examine whether there is a three-way interaction between treatment, discernment, and whether a headline was one of the first 7 headlines shown to a participant as part of the study (coded as a binary variable, where a value of 1 means that a headline was one of the first 7 headlines shown to a participant and a value of 2 means that it was among the last 23 headlines). When comparing the treatment effect in the first 7 headlines to the last 23 headlines, we find a significant three-way interaction between treatment, discernment and headline order (*β* = -0.204, 95%CI [-0.37, -0.040], *F*(7,47490) = 3.50, *p_exploratory_* = 0.015)^[[1]](#footnote-1)^ so that the treatment effect is significant for the first 7 headlines (*β* = 0.23, 95%CI [0.088, 0.37], *F*(3,11081) = 6.53, *p_exploratory_* = 0.002), but not the last 23 headlines (*β* = 0.027, 95%CI [-0.043, 0.098], *F*(3,36409) = 2.27, *p_exploratory_* = 0.448). This difference in the treatment effect conferred by the accuracy nudge observed in the first 7 versus the last 23 headlines in the pooled sample (*N* = 1,583) and the second round of data collection (*n* = 882) is visualized in Figure S3 and reported in Table S10.

| 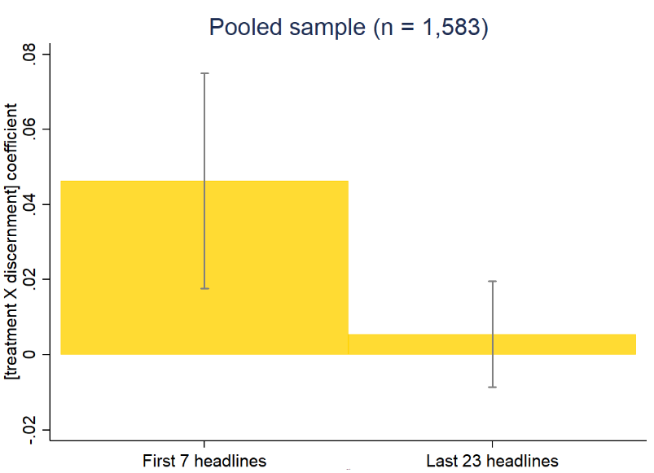 | 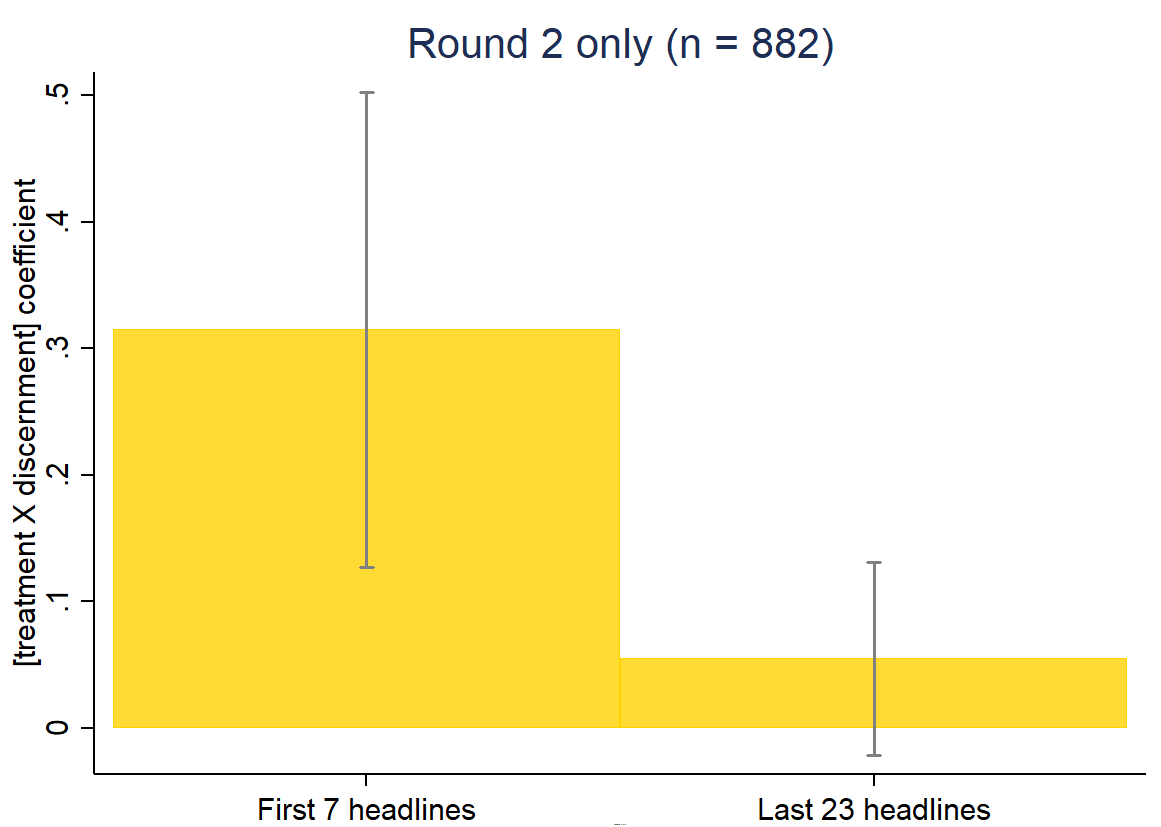 |
| --- | --- |

*Figure S3*. Bar graphs of the treatment x sharing discernment coefficient by headline order (first 7 headlines vs last 23 headlines) for the pooled sample (*n* = 1,583, left panel) and second round (*n* = 882, second panel). A higher coefficient represents a larger effect of the accuracy nudge on sharing discernment. Error bars represent standard errors.

Our exploratory findings thus offer some initial support for the idea that the treatment effect conferred by the accuracy nudge is by far the most prominent in the first few headlines that participants are asked to rate and appears to taper off soon thereafter. This is in line with previous research into the decay of priming effects (Branigan et al., 1999; Trammell & Valdes, 1992). However, although we did note in our pre-registration that we would examine potential decay effects, we did not pre-register an analysis plan, so we refrain from drawing firm conclusions about whether accuracy nudges are subject to rapid decay, particularly as the gap between treatment and control could also get smaller with trial number if baseline sharing decreases over time in the control group. In addition, decay patterns in survey data might differ from how accuracy nudges behave over time on social media platforms (see Pennycook et al., 2021). We therefore encourage future research to look into this question more explicitly using longitudinal designs, as the potential implications of decay for the implementation of accuracy nudges in social media environments are substantial.

**Table S1**

*Quota composition*

| **Category** | **Value** | **%** | **N_target,Stage1_** | **N_target,Stage2_** |
| --- | --- | --- | --- | --- |
| Age/Gender | Males 18-24 | 6.70% | 47 | 59 |
|  | Males 25-34 | 8.80% | 62 | 78 |
|  | Males 35-44 | 8.70% | 61 | 77 |
|  | Males 45-54 | 9.40% | 66 | 83 |
|  | Males 55-64 | 7.50% | 52 | 66 |
|  | Males 65-75 | 7.40% | 52 | 65 |
|  | Females 18-24 | 6.40% | 45 | 56 |
|  | Females 25-34 | 8.71% | 61 | 77 |
|  | Females 35-44 | 8.80% | 62 | 78 |
|  | Females 45-54 | 9.75% | 68 | 86 |
|  | Females 55-64 | 8.05% | 56 | 71 |
|  | Females 65-75 | 9.80% | 69 | 86 |
| Geographic Region | Northeast | 20.00% | 140 | 176 |
|  | Midwest | 20.00% | 140 | 176 |
|  | South | 34.00% | 239 | 300 |
|  | West | 26.00% | 182 | 230 |
| Racial Background | White/Caucasian | 72.00% | 504 | 635 |
|  | African American | 13.00% | 91 | 114 |
|  | Native American | 1.00% | 7 | 8 |
|  | Asian/Pacific Islander | 6.00% | 42 | 52 |
|  | Mixed Race | 3.30% | 23 | 29 |
|  | Other/prefer not to say | 5.00% | 34 | 44 |

**Table S2**

*Sample composition for data collection stages 1 and 2.*

|  | **First round (*n* = 701)** | | | **Pooled sample (*N* = 1,583)** | | | |
| --- | --- | --- | --- | --- | --- | --- | --- |
| **Variable** | *N* | *%* | *Cum. %* |  | *N* | *%* | *Cum. %* |
|  |  |  |  |  |  |  |  |
| **Gender** | | | |  |  | | |
| Female | 360 | 51.4 % | 51.4 % |  | 814 | 51.4 % | 51.4 % |
| Male | 338 | 48.2 % | 99.6 % |  | 764 | 48.3 % | 99.7 % |
| Trans/Non-Binary | 1 | 0.1 % | 99.7 % |  | 2 | 0.1 % | 99.8 % |
| Transgender Female | 1 | 0.1 % | 99.9 % |  | 1 | 0.1 % | 99.9 % |
| Transgender Male | 1 | 0.1 % | 100.0 % |  | 2 | 0.1 % | 100.0 % |
|  |  |  |  |  |  |  |  |
| **Geographic region** | | | |  |  | | |
| Mid-West | 140 | 20.0 % | 20.0 % |  | 318 | 20.1 % | 20.1 % |
| Northeast | 140 | 20.0 % | 39.9 % |  | 318 | 20.1 % | 40.2 % |
| South | 239 | 34.1 % | 74.0 % |  | 544 | 34.4 % | 74.5 % |
| West | 182 | 26.0 % | 100.0 % |  | 403 | 25.5 % | 100.0 % |
|  | | | |  |  | | |
| **Racial background** | | | |  |  | | |
| White/Caucasian | 526 | 75.0 % | 75.0 % |  | 1188 | 75.0 % | 75.0 % |
| African American | 95 | 13.6 % | 88.6 % |  | 214 | 13.5 % | 88.6 % |
| Asian/Pacific Islander | 42 | 6.0 % | 94.6 % |  | 95 | 6.0 % | 94.6 % |
| Native American | 7 | 1.0 % | 95.6 % |  | 16 | 1.0 % | 95.6 % |
| Mixed Race | 24 | 3.4 % | 99.0 % |  | 54 | 3.4 % | 99.0 % |
| Other/Prefer not to say | 7 | 1.0 % | 100.0 % |  | 16 | 1.0 % | 100.0 % |
|  |  |  |  |  |  |  |  |
| **Party affiliation** |  |  |  |  |  |  |  |
| Strongly Democratic | 111 | 15.8 % | 15.8 % |  | 266 | 16.8 % | 16.8 % |
| Democratic | 125 | 17.8 % | 33.7 % |  | 278 | 17.6 % | 34.4 % |
| Lean Democratic | 164 | 23.4 % | 57.1 % |  | 360 | 22.7 % | 57.1 % |
| Lean Republican | 145 | 20.7 % | 77.7 % |  | 325 | 20.5 % | 77.6 % |
| Republican | 75 | 10.7 % | 88.4 % |  | 171 | 10.8 % | 88.4 % |
| Strongly Republican | 81 | 11.6 % | 100.0 % |  | 183 | 11.6 % | 100.0 % |
|  | | | |  |  | | |
| **Political ideology - social issues** | | | |  |  | | |
| Strongly Liberal | 107 | 15.3 % | 15.3 % |  | 248 | 15.7 % | 15.7 % |
| Somewhat Liberal | 147 | 21.0 % | 36.2 % |  | 333 | 21.0 % | 36.7 % |
| Moderate | 259 | 36.9 % | 73.2 % |  | 565 | 35.7 % | 72.4 % |
| Somewhat Conservative | 99 | 14.1 % | 87.3 % |  | 239 | 15.1 % | 87.5 % |
| Strongly Conservative | 89 | 12.7 % | 100.0 % |  | 198 | 12.5 % | 100.0 % |
|  | | | |  |  | | |
| **Political ideology - economic issues** | | | |  |  | | |
| Strongly Liberal | 82 | 11.7 % | 11.7 % |  | 198 | 12.5 % | 12.5 % |
| Somewhat Liberal | 125 | 17.8 % | 29.5 % |  | 277 | 17.5 % | 30.0 % |
| Moderate | 263 | 37.5 % | 67.0 % |  | 577 | 36.4 % | 66.5 % |
| Somewhat Conservative | 124 | 17.7 % | 84.7 % |  | 297 | 18.8 % | 85.2 % |
| Strongly Conservative | 107 | 15.3 % | 100.0 % |  | 234 | 14.8 % | 100.0 % |
|  | | | |  |  | | |
| **How often do you proactively check the news regarding COVID-19?** | | | |  |  | | |
| Never | 20 | 2.9 % | 2.9 % |  | 35 | 2.2 % | 2.2 % |
| Rarely | 64 | 9.1 % | 12.0 % |  | 147 | 9.3 % | 11.5 % |
| Sometimes | 170 | 24.3 % | 36.2 % |  | 391 | 24.7 % | 36.2 % |
| Often | 273 | 38.9 % | 75.2 % |  | 592 | 37.4 % | 73.6 % |
| Very Often | 174 | 24.8 % | 100.0 % |  | 418 | 26.4 % | 100.0 % |
|  | | | |  |  | | |
| **Number of attention checks passed** | |  |  |  |  |  |  |
| 0 | 7 | 1.0 % | 1.0 % |  | 30 | 1.9 % | 1.9 % |
| 1 | 129 | 18.4 % | 19.4 % |  | 322 | 20.3 % | 22.2 % |
| 2 | 367 | 52.4 % | 71.8 % |  | 788 | 49.8 % | 72.0 % |
| 3 | 198 | 28.2 % | 100.0 % |  | 443 | 28.0 % | 100.0 % |
|  |  |  |  |  |  |  |  |
| **Continuous variables** |  | *M* | *SD* |  |  | *M* | *SD* |
|  |  |  |  |  |  |  |  |
| **Age** |  | 45.6 | 16.2 |  |  | 45.4 | 16.3 |
| **Years of education** |  | 14.2 | 4.26 |  |  | 14.3 | 4.1 |
| **Science knowledge** |  | 0.602 | 0.212 |  |  | 0.586 | 0.21 |
| **CRT score** |  | 0.363 | 0.289 |  |  | 0.356 | 0.282 |
| **MMS** |  | 4.48 | 0.97 |  |  | 4.56 | 0.974 |
| **Level of concern about COVID** |  | 73.3 | 27.8 |  |  | 73.8 | 26.9 |

**Table S3**

*Items descriptive statistics (stage 2).*

| **Variable** | **Group** | **N** | **Mean** | **Median** | **SD** | **SE** |
| --- | --- | --- | --- | --- | --- | --- |
| Avg_Fake | Control | 793 | 3.04 | 3.07 | 1.41 | 0.050 |
|  | Treatment | 790 | 2.89 | 2.87 | 1.40 | 0.050 |
| Avg_Real | Control | 793 | 3.23 | 3.27 | 1.32 | 0.047 |
|  | Treatment | 790 | 3.15 | 3.27 | 1.34 | 0.048 |
| Discernment | Control | 793 | 0.19 | 0.07 | 0.69 | 0.025 |
|  | Treatment | 790 | 0.26 | 0.07 | 0.79 | 0.028 |
| Fake1_1 | Control | 793 | 2.77 | 2.00 | 1.68 | 0.060 |
|  | Treatment | 790 | 2.73 | 2.00 | 1.68 | 0.060 |
| Fake1_2 | Control | 793 | 3.17 | 3.00 | 1.88 | 0.067 |
|  | Treatment | 790 | 3.01 | 3.00 | 1.88 | 0.067 |
| Fake1_3 | Control | 793 | 2.75 | 2.00 | 1.73 | 0.061 |
|  | Treatment | 790 | 2.66 | 2.00 | 1.77 | 0.063 |
| Fake1_4 | Control | 793 | 3.04 | 3.00 | 1.83 | 0.065 |
|  | Treatment | 790 | 2.77 | 2.00 | 1.77 | 0.063 |
| Fake1_5 | Control | 793 | 2.74 | 2.00 | 1.80 | 0.064 |
|  | Treatment | 790 | 2.66 | 2.00 | 1.76 | 0.063 |
| Fake1_6 | Control | 793 | 3.61 | 4.00 | 1.87 | 0.066 |
|  | Treatment | 790 | 3.47 | 4.00 | 1.88 | 0.067 |
| Fake1_7 | Control | 793 | 3.06 | 3.00 | 1.75 | 0.062 |
|  | Treatment | 790 | 2.90 | 3.00 | 1.74 | 0.062 |
| Fake1_8 | Control | 793 | 3.04 | 3.00 | 1.77 | 0.063 |
|  | Treatment | 790 | 2.87 | 3.00 | 1.77 | 0.063 |
| Fake1_9 | Control | 793 | 3.15 | 3.00 | 1.76 | 0.062 |
|  | Treatment | 790 | 3.05 | 3.00 | 1.82 | 0.065 |
| Fake1_10 | Control | 793 | 3.08 | 3.00 | 1.83 | 0.065 |
|  | Treatment | 790 | 2.87 | 3.00 | 1.82 | 0.065 |
| Fake1_11 | Control | 793 | 2.43 | 1.00 | 1.77 | 0.063 |
|  | Treatment | 790 | 2.30 | 1.00 | 1.72 | 0.061 |
| Fake1_12 | Control | 793 | 2.99 | 3.00 | 1.73 | 0.061 |
|  | Treatment | 790 | 2.86 | 3.00 | 1.75 | 0.062 |
| Fake1_13 | Control | 793 | 3.32 | 3.00 | 1.78 | 0.063 |
|  | Treatment | 790 | 3.11 | 3.00 | 1.80 | 0.064 |
| Fake1_14 | Control | 793 | 3.13 | 3.00 | 1.94 | 0.069 |
|  | Treatment | 790 | 2.98 | 3.00 | 1.94 | 0.069 |
| Fake1_15 | Control | 793 | 3.31 | 3.00 | 1.88 | 0.067 |
|  | Treatment | 790 | 3.12 | 3.00 | 1.85 | 0.066 |
| Real1_1 | Control | 793 | 3.86 | 4.00 | 1.75 | 0.062 |
|  | Treatment | 790 | 3.74 | 4.00 | 1.73 | 0.062 |
| Real1_2 | Control | 793 | 2.90 | 3.00 | 1.76 | 0.063 |
|  | Treatment | 790 | 2.77 | 2.00 | 1.73 | 0.062 |
| Real1_3 | Control | 793 | 3.35 | 3.00 | 1.78 | 0.063 |
|  | Treatment | 790 | 3.18 | 3.00 | 1.80 | 0.064 |
| Real1_4 | Control | 793 | 2.91 | 2.00 | 1.84 | 0.065 |
|  | Treatment | 790 | 2.76 | 2.00 | 1.80 | 0.064 |
| Real1_5 | Control | 793 | 3.07 | 3.00 | 1.72 | 0.061 |
|  | Treatment | 790 | 3.12 | 3.00 | 1.80 | 0.064 |
| Real1_6 | Control | 793 | 3.19 | 3.00 | 1.69 | 0.060 |
|  | Treatment | 790 | 3.19 | 3.00 | 1.68 | 0.060 |
| Real1_7 | Control | 793 | 3.10 | 3.00 | 1.69 | 0.060 |
|  | Treatment | 790 | 2.95 | 3.00 | 1.71 | 0.061 |
| Real1_8 | Control | 793 | 3.67 | 4.00 | 1.80 | 0.064 |
|  | Treatment | 790 | 3.53 | 4.00 | 1.80 | 0.064 |
| Real1_9 | Control | 793 | 2.82 | 3.00 | 1.77 | 0.063 |
|  | Treatment | 790 | 2.92 | 3.00 | 1.81 | 0.064 |
| Real1_10 | Control | 793 | 2.88 | 3.00 | 1.72 | 0.061 |
|  | Treatment | 790 | 2.94 | 3.00 | 1.77 | 0.063 |
| Real1_11 | Control | 793 | 2.67 | 2.00 | 1.77 | 0.063 |
|  | Treatment | 790 | 2.51 | 2.00 | 1.73 | 0.061 |
| Real1_12 | Control | 793 | 3.58 | 4.00 | 1.72 | 0.061 |
|  | Treatment | 790 | 3.50 | 4.00 | 1.70 | 0.061 |
| Real1_13 | Control | 793 | 3.34 | 3.00 | 1.81 | 0.064 |
|  | Treatment | 790 | 3.32 | 4.00 | 1.89 | 0.067 |
| Real1_14 | Control | 793 | 3.25 | 3.00 | 1.78 | 0.063 |
|  | Treatment | 790 | 3.17 | 3.00 | 1.78 | 0.063 |
| Real1_15 | Control | 793 | 3.78 | 4.00 | 1.67 | 0.059 |
|  | Treatment | 790 | 3.67 | 4.00 | 1.69 | 0.060 |

**Table S4**

*Independent samples & Bayesian t-tests for discernment, false & real headlines, stage 2.*

|  |  | **Statistic** | **Error %** | **df** | **p** | **M_diff_** | **M_diff_ 95% CI** | **d** | **Cohen’s d 95% CI** |
| --- | --- | --- | --- | --- | --- | --- | --- | --- | --- |
| Avg_Fake | Student's t | 2.1128 |  | 1581 | 0.035 | 0.149 | [0.01067 - 0.28727] | 0.11 | [0.00751 - 0.20483] |
|  | Bayes factor₁₀* | 1.948 | 0.003 |  |  |  |  |  |  |
| Avg_Real | Student's t | 1.1098 |  | 1581 | 0.267 | 0.074 | [-0.05685 - 0.20501] | 0.06 | [-0.04279 - 0.15433] |
|  | Bayes factor₁₀ | 0.732 | 5.152e -7 |  |  |  |  |  |  |
| Discernment | Student's t | -2.0129 |  | 1581 | 0.044 | -0.075 | [-0.14786 - -0.00191] | -0.10 | [-0.1998 - -0.0025] |
|  | Bayes factor₁₀ | 1.705 | 7.148e -6 |  |  |  |  |  |  |
| Fake1_1 | Student's t | 0.4905 |  | 1581 | 0.624 | 0.041 | [-0.12411 - 0.20689] | 0.02 | [-0.07388 - 0.12318] |
|  | Bayes factor₁₀ | 0.559 | 0.008 |  |  |  |  |  |  |
| Fake1_2 | Student's t | 1.6858 |  | 1581 | 0.092 | 0.159 | [-0.02597 - 0.34371] | 0.08 | [-0.0139 - 0.18333] |
|  | Bayes factor₁₀ | 1.17 | 3.658e -6 |  |  |  |  |  |  |
| Fake1_3 | Student's t | 1.0327 |  | 1581 | 0.302 | 0.091 | [-0.08167 - 0.2633] | 0.05 | [-0.04666 - 0.15045] |
|  | Bayes factor₁₀ | 0.699 | 3.958e -7 |  |  |  |  |  |  |
| Fake1_4 | Student's t | 3.0116 |  | 1581 | 0.003 | 0.272 | [0.09484 - 0.44915] | 0.15 | [0.05253 - 0.25014] |
|  | Bayes factor₁₀ | 9.929 | 2.160e -6 |  |  |  |  |  |  |
| Fake1_5 | Student's t | 0.9578 |  | 1581 | 0.338 | 0.086 | [-0.0899 - 0.26147] | 0.05 | [-0.05042 - 0.14668] |
|  | Bayes factor₁₀ | 0.671 | 2.175e -6 |  |  |  |  |  |  |
| Fake1_6 | Student's t | 1.5471 |  | 1581 | 0.122 | 0.146 | [-0.03904 - 0.3306] | 0.08 | [-0.02085 - 0.17634] |
|  | Bayes factor₁₀ | 1.023 | 2.153e -6 |  |  |  |  |  |  |
| Fake1_7 | Student's t | 1.8423 |  | 1581 | 0.066 | 0.162 | [-0.01047 - 0.33409] | 0.09 | [-0.00605 - 0.19121] |
|  | Bayes factor₁₀ | 1.386 | 6.293e -6 |  |  |  |  |  |  |
| Fake1_8 | Student's t | 1.9073 |  | 1581 | 0.057 | 0.169 | [-0.00482 - 0.34379] | 0.10 | [-0.00279 - 0.19448] |
|  | Bayes factor₁₀ | 1.496 | 8.931e -6 |  |  |  |  |  |  |
| Fake1_9 | Student's t | 1.0512 |  | 1581 | 0.293 | 0.094 | [-0.08173 - 0.2705] | 0.05 | [-0.04573 - 0.15138] |
|  | Bayes factor₁₀ | 0.707 | 1.167e -7 |  |  |  |  |  |  |
| Fake1_10 | Student's t | 2.2324 |  | 1581 | 0.026 | 0.205 | 0.02485 - 0.38469] | 0.11 | [0.0135 - 0.21086] |
|  | Bayes factor₁₀ | 2.311 | 5.733e -6 |  |  |  |  |  |  |
| Fake1_11 | Student's t | 1.4563 |  | 1581 | 0.145 | 0.127 | [-0.04422 - 0.29919] | 0.07 | [-0.0254 - 0.17177] |
|  | Bayes factor₁₀ | 0.944 | 5.176e -7 |  |  |  |  |  |  |
| Fake1_12 | Student's t | 1.4614 |  | 1581 | 0.144 | 0.128 | [-0.04376 - 0.29954] | 0.07 | [-0.02515 - 0.17203] |
|  | Bayes factor₁₀ | 0.948 | 5.517e -7 |  |  |  |  |  |  |
| Fake1_13 | Student's t | 2.335 |  | 1581 | 0.02 | 0.210 | 0.03362 - 0.38672] | 0.12 | [0.01864 - 0.21603] |
|  | Bayes factor₁₀ | 2.704 | 0.004 |  |  |  |  |  |  |
| Fake1_14 | Student's t | 1.541 |  | 1581 | 0.124 | 0.150 | [-0.04097 - 0.34124] | 0.08 | [-0.02116 - 0.17604] |
|  | Bayes factor₁₀ | 1.017 | 1.996e -6 |  |  |  |  |  |  |
| Fake1_15 | Student's t | 2.0678 |  | 1581 | 0.039 | 0.194 | 0.00996 - 0.37756] | 0.10 | [0.00526 - 0.20257] |
|  | Bayes factor₁₀ | 1.833 | 1.271e -4 |  |  |  |  |  |  |
| Real1_1 | Student's t | 1.3807 |  | 1581 | 0.168 | 0.121 | [-0.0508 - 0.29235] | 0.07 | [-0.0292 - 0.16797] |
|  | Bayes factor₁₀ | 0.887 | 6.558e -7 |  |  |  |  |  |  |
| Real1_2 | Student's t | 1.5031 |  | 1581 | 0.133 | 0.132 | [-0.04025 - 0.30427] | 0.08 | [-0.02306 - 0.17413] |
|  | Bayes factor₁₀ | 0.983 | 1.135e -6 |  |  |  |  |  |  |
| Real1_3 | Student's t | 1.8821 |  | 1581 | 0.06 | 0.170 | [-0.00715 - 0.34629] | 0.09 | [-0.00406 - 0.19321] |
|  | Bayes factor₁₀ | 1.452 | 8.241e -6 |  |  |  |  |  |  |
| Real1_4 | Student's t | 1.638 |  | 1581 | 0.102 | 0.150 | [-0.02956 - 0.329] | 0.08 | [-0.01629 - 0.18092] |
|  | Bayes factor₁₀ | 1.116 | 0.005 |  |  |  |  |  |  |
| Real1_5 | Student's t | -0.5046 |  | 1581 | 0.614 | -0.045 | [-0.21788 - 0.12871] | -0.03 | [-0.12389 - 0.07317] |
|  | Bayes factor₁₀ | 0.561 | 0.011 |  |  |  |  |  |  |
| Real1_6 | Student's t | 0.0216 |  | 1581 | 0.983 | 0.002 | [-0.16403 - 0.16768] | 0.00 | [-0.09744 - 0.09961] |
|  | Bayes factor₁₀ | 0.525 | 0.012 |  |  |  |  |  |  |
| Real1_7 | Student's t | 1.7152 |  | 1581 | 0.087 | 0.146 | [-0.02103 - 0.31397] | 0.09 | [-0.01242 - 0.18481] |
|  | Bayes factor₁₀ | 1.206 | 3.104e -6 |  |  |  |  |  |  |
| Real1_8 | Student's t | 1.5637 |  | 1581 | 0.118 | 0.142 | [-0.03606 - 0.31956] | 0.08 | [-0.02002 - 0.17718] |
|  | Bayes factor₁₀ | 1.039 | 2.581e -6 |  |  |  |  |  |  |
| Real1_9 | Student's t | -1.0904 |  | 1581 | 0.276 | -0.098 | [-0.27441 - 0.07832] | -0.05 | [-0.15336 - 0.04376] |
|  | Bayes factor₁₀ | 0.724 | 2.673e -7 |  |  |  |  |  |  |
| Real1_10 | Student's t | -0.7032 |  | 1581 | 0.482 | -0.062 | [-0.23334 - 0.11018] | -0.04 | [-0.13388 - 0.0632] |
|  | Bayes factor₁₀ | 0.598 | 2.115e -6 |  |  |  |  |  |  |
| Real1_11 | Student's t | 1.7869 |  | 1581 | 0.074 | 0.157 | [-0.01533 - 0.32924] | 0.09 | [-0.00882 - 0.18842] |
|  | Bayes factor₁₀ | 1.303 | 3.225e -6 |  |  |  |  |  |  |
| Real1_12 | Student's t | 0.8877 |  | 1581 | 0.375 | 0.076 | [-0.09228 - 0.24485] | 0.04 | [-0.05394 - 0.14316] |
|  | Bayes factor₁₀ | 0.648 | 2.586e -6 |  |  |  |  |  |  |
| Real1_13 | Student's t | 0.2721 |  | 1581 | 0.786 | 0.025 | [-0.15692 - 0.20747] | 0.01 | [-0.08485 - 0.1122] |
|  | Bayes factor₁₀ | 0.535 | 0.004 |  |  |  |  |  |  |
| Real1_14 | Student's t | 0.964 |  | 1581 | 0.335 | 0.086 | [-0.08938 - 0.26214] | 0.05 | [-0.05011 - 0.14699] |
|  | Bayes factor₁₀ | 0.673 | 2.039e -6 |  |  |  |  |  |  |
| Real1_15 | Student's t | 1.2865 |  | 1581 | 0.198 | 0.108 | [-0.05689 - 0.27376] | 0.06 | [-0.03393 - 0.16322] |
|  | Bayes factor₁₀ | 0.825 | 9.102e -7 |  |  |  |  |  |  |
| ᵃ Levene's test is significant (*p* < .05), suggesting a violation of the assumption of equal variances. | | | | | | | |  |  |
| * The prior is described by a Cauchy distribution centred around 0 and with a width parameter of 0.05, corresponding to a 79% chance that the effect size lies between -0.15 and 0.15; this is in line with the effect sizes reported in the target study reported by Pennycook et al. (2020). | | | | | | | | | |

**Table S5**

*Stage 1, linear regression (ivreg2) with robust standard errors clustered on participants and headlines (column 1); with standardized coefficients (column 2); and high-dimensional fixed effects (column 3).*

|  | (1) | (2) | (3) |
| --- | --- | --- | --- |
| **Variables** | rating | rating | rating |
|  |  |  |  |
| rating |  |  |  |
|  | ( - ) | ( - ) | ( - ) |
|  |  |  |  |
| real | 0.0482* | 0.0482*** | 0.0482* |
|  | (-0.000271 - 0.0967) | (0.0345 - 0.0620) | (-0.00325 - 0.0997) |
|  | 0.0247 | 0.00701 | 0.0252 |
| treatment | -0.00555 | -0.00555 | -0.00555 |
|  | (-0.0451 - 0.0340) | (-0.0192 - 0.00813) | (-0.0476 - 0.0365) |
|  | 0.0202 | 0.00698 | 0.0205 |
| realxtreatment | 0.00462 | 0.00462 | 0.00462 |
|  | (-0.0164 - 0.0257) | (-0.0147 - 0.0240) | (-0.0177 - 0.0270) |
|  | 0.0107 | 0.00987 | 0.0109 |
| Constant | 0.376*** | 0.376*** | 0.376*** |
|  | (0.336 - 0.416) | (0.366 - 0.386) | (0.334 - 0.418) |
|  | 0.0204 | 0.00496 | 0.0207 |
|  |  |  |  |
| Observations | 21,030 | 21,030 | 21,030 |
| R-squared | 0.005 | 0.005 | 0.005 |

Robust ci in parentheses, *** *p*<0.01, ** *p*<0.05, * *p*<0.1.

**Table S6**

*Stage 2, linear regression (ivreg2) with robust standard errors clustered on participants and headlines (column 1); with standardized coefficients (column 2); and high-dimensional fixed effects (column 3).*

|  | (1) | (2) | (3) |
| --- | --- | --- | --- |
| **Variables** | rating | rating | rating |
|  |  |  |  |
| rating |  |  |  |
|  | ( - ) | ( - ) | ( - ) |
|  |  |  |  |
| real | 0.0373 | 0.0373*** | 0.0373 |
|  | (-0.00833 - 0.0830) | (0.0282 - 0.0465) | (-0.0111 - 0.0858) |
|  | 0.0233 | 0.00468 | 0.0237 |
| treatment | -0.0298** | -0.0298*** | -0.0298** |
|  | (-0.0564 - -0.00315) | (-0.0390 - -0.0206) | (-0.0581 - -0.00151) |
|  | 0.0136 | 0.00468 | 0.0138 |
| realxtreatment | 0.0150** | 0.0150** | 0.0150** |
|  | (0.00271 - 0.0272) | (0.00200 - 0.0280) | (0.00196 - 0.0280) |
|  | 0.00626 | 0.00662 | 0.00637 |
| Constant | 0.408*** | 0.408*** | 0.408*** |
|  | (0.374 - 0.441) | (0.401 - 0.414) | (0.372 - 0.443) |
|  | 0.0171 | 0.00331 | 0.0174 |
|  |  |  |  |
| Observations | 47,490 | 47,490 | 47,490 |
| R-squared | 0.005 | 0.005 | 0.005 |

Robust ci in parentheses, *** *p*<0.01, ** *p*<0.05, * *p*<0.1.

**Table S7**

*Stage 2, linear regression (ivreg2) with robust standard errors clustered on participants and headlines, for different levels of attentiveness.*

|  | **Attention > -1** | **Attention > 0** | **Attention > 1** | **Attention > 2** |
| --- | --- | --- | --- | --- |
| **Variables** | rating | rating | rating | rating |
|  |  |  |  |  |
| rating |  |  |  |  |
|  | ( - ) | ( - ) | ( - ) | ( - ) |
|  |  |  |  |  |
| real | 0.0373 | 0.0379 | 0.0456* | 0.0635** |
|  | (-0.00833 - 0.0830) | (-0.00823 - 0.0841) | (-0.00296 - 0.0942) | (0.00548 - 0.122) |
|  | 0.0233 | 0.0236 | 0.0248 | 0.0296 |
| treatment | -0.0298** | -0.0320** | -0.0299** | -0.0506** |
|  | (-0.0564 - -0.00315) | (-0.0585 - -0.00546) | (-0.0590 - -0.000741) | (-0.0957 - -0.00558) |
|  | 0.0136 | 0.0135 | 0.0149 | 0.0230 |
| realxtreatment | 0.0150** | 0.0151** | 0.0135* | 0.00646 |
|  | (0.00271 - 0.0272) | (0.00241 - 0.0278) | (-0.000843 - 0.0279) | (-0.0205 - 0.0334) |
|  | 0.00626 | 0.00647 | 0.00734 | 0.0138 |
| Constant | 0.408*** | 0.402*** | 0.377*** | 0.331*** |
|  | (0.374 - 0.441) | (0.368 - 0.435) | (0.341 - 0.413) | (0.285 - 0.378) |
|  | 0.0171 | 0.0171 | 0.0183 | 0.0237 |
|  |  |  |  |  |
| Observations | 47,490 | 46,590 | 36,930 | 13,290 |
| R-squared | 0.005 | 0.005 | 0.007 | 0.014 |

Robust ci in parentheses. *p*-value for realxtreatment for attention > 1 = 0.065. *** *p*<0.01, ** *p*<0.05, * *p*<0.1.

**Table S8**

*Stage 2, interaction effects with political partisanship, CRT score, scientific knowledge and MMS.*

|  | **Political partisanship** | **CRT** | **Science knowledge** | **MMS** |
| --- | --- | --- | --- | --- |
| **Variables** | rating | rating | rating | rating |
|  |  |  |  |  |
| rating |  |  |  |  |
|  | ( - ) | ( - ) | ( - ) | ( - ) |
|  |  |  |  |  |
| real | 0.0366 | 0.0367 | 0.0362 | 0.0371 |
|  | (-0.00875 - 0.0820) | (-0.00878 - 0.0822) | (-0.00916 - 0.0816) | (-0.00864 - 0.0829) |
|  | 0.0232 | 0.0232 | 0.0232 | 0.0233 |
| treatment | -0.0307** | -0.0365*** | -0.0336** | -0.0355*** |
|  | (-0.0573 - -0.00415) | (-0.0623 - -0.0108) | (-0.0599 - -0.00735) | (-0.0594 - -0.0115) |
|  | 0.0136 | 0.0131 | 0.0134 | 0.0122 |
| zdemrep | 0.00946 |  |  |  |
|  | (-0.0155 - 0.0344) |  |  |  |
|  | 0.0127 |  |  |  |
| realxtreatment | 0.0165*** | 0.0160** | 0.0172*** | 0.0148** |
|  | (0.00479 - 0.0283) | (0.00347 - 0.0284) | (0.00498 - 0.0294) | (0.00183 - 0.0277) |
|  | 0.00599 | 0.00637 | 0.00622 | 0.00660 |
| realXzdemrep | -0.0323*** |  |  |  |
|  | (-0.0558 - -0.00871) |  |  |  |
|  | 0.0120 |  |  |  |
| treatmentXzdemrep | 0.0242* |  |  |  |
|  | (-0.00439 - 0.0527) |  |  |  |
|  | 0.0146 |  |  |  |
| realXtreatmentxzdemrep | -0.00826 |  |  |  |
|  | (-0.0194 - 0.00293) |  |  |  |
|  | 0.00571 |  |  |  |
| zcrt |  | -0.0652*** |  |  |
|  |  | (-0.0830 - -0.0473) |  |  |
|  |  | 0.00912 |  |  |
| realXzcrt |  | 0.0132*** |  |  |
|  |  | (0.00371 - 0.0228) |  |  |
|  |  | 0.00486 |  |  |
| treatmentXzcrt |  | -0.0177 |  |  |
|  |  | (-0.0427 - 0.00725) |  |  |
|  |  | 0.0128 |  |  |
| realXtreatmentxzcrt |  | -0.00492 |  |  |
|  |  | (-0.0165 - 0.00671) |  |  |
|  |  | 0.00593 |  |  |
| zSK |  |  | -0.0471*** |  |
|  |  |  | (-0.0683 - -0.0260) |  |
|  |  |  | 0.0108 |  |
| realXzSK |  |  | 0.0296*** |  |
|  |  |  | (0.0162 - 0.0431) |  |
|  |  |  | 0.00686 |  |
| treatmentXzSK |  |  | -0.00932 |  |
|  |  |  | (-0.0357 - 0.0170) |  |
|  |  |  | 0.0134 |  |
| realXtreatmentxzSK |  |  | -6.87e-05 |  |
|  |  |  | (-0.0136 - 0.0134) |  |
|  |  |  | 0.00689 |  |
| zMMS |  |  |  | 0.132*** |
|  |  |  |  | (0.114 - 0.151) |
|  |  |  |  | 0.00941 |
| realXzMMS |  |  |  | -0.00900** |
|  |  |  |  | (-0.0174 - -0.000619) |
|  |  |  |  | 0.00428 |
| treatmentXzMMS |  |  |  | -0.0183 |
|  |  |  |  | (-0.0440 - 0.00745) |
|  |  |  |  | 0.0131 |
| realXtreatmentxzMMS |  |  |  | 0.0270*** |
|  |  |  |  | (0.0138 - 0.0401) |
|  |  |  |  | 0.00671 |
| Constant | 0.408*** | 0.411*** | 0.409*** | 0.411*** |
|  | (0.375 - 0.441) | (0.377 - 0.444) | (0.376 - 0.443) | (0.378 - 0.443) |
|  | 0.0170 | 0.0169 | 0.0170 | 0.0165 |
| Observations | 47,490 | 47,490 | 47,490 | 47,490 |
| R-squared | 0.008 | 0.041 | 0.017 | 0.125 |

Robust ci in parentheses, *** *p*<0.01, ** *p*<0.05, * *p*<0.1.

**Table S9**

*Stage 2, main analysis with different levels of accuracy difference from study 1.*

|  | **Acc. Diff. < 0.1** | **Acc. Diff <0.05** | **Acc. Diff. < 0.025** |
| --- | --- | --- | --- |
| **Variables** | rating | rating | rating |
|  |  |  |  |
| rating |  |  |  |
|  | ( - ) | ( - ) | ( - ) |
|  |  |  |  |
| real | 0.0437* | 0.00917 | -0.0728*** |
|  | (-0.00420 - 0.0915) | (-0.0493 - 0.0677) | (-0.116 - -0.0295) |
|  | 0.0244 | 0.0299 | 0.0221 |
| treatment | -0.0297** | -0.0329** | -0.0336*** |
|  | (-0.0563 - -0.00303) | (-0.0596 - -0.00615) | (-0.0580 - -0.00923) |
|  | 0.0136 | 0.0136 | 0.0124 |
| realxtreatment | 0.0159** | 0.0217*** | 0.0459 |
|  | (0.00357 - 0.0283) | (0.00825 - 0.0351) | ( - ) |
|  | 0.00630 | 0.00686 |  |
| Constant | 0.406*** | 0.414*** | 0.449*** |
|  | (0.371 - 0.441) | (0.372 - 0.456) | (0.399 - 0.499) |
|  | 0.0178 | 0.0214 | 0.0256 |
|  |  |  |  |
| Observations | 44,324 | 30,077 | 7,915 |
| R-squared | 0.006 | 0.002 | 0.005 |

Robust ci in parentheses, *** *p*<0.01, ** *p*<0.05, * *p*<0.1.

**Table S10**

*Linear regression with robust standard errors clustered on participants and headlines, with headline order (the first 3 headlines versus the last 27 headlines shown to each participant) included, for the full sample (column 1), stage 1 (column 2) and stage 2 (column 3).*

|  | **Pooled sample** | **Stage 1** | **Stage 2** |
| --- | --- | --- | --- |
| **Variables** | rating | rating | rating |
|  |  |  |  |
| real | 0.0951 | 0.187 | 0.0170 |
|  | (-0.279 - 0.469) | (-0.415 - 0.790) | (-0.356 - 0.390) |
| treatment | -0.190 | 0.119 | -0.440** |
|  | (-0.505 - 0.126) | (-0.258 - 0.495) | (-0.868 - -0.0116) |
| binorder | 0.0396 | 0.0384 | 0.0383 |
|  | (-0.0466 - 0.126) | (-0.136 - 0.212) | (-0.0708 - 0.147) |
| realxtreatment | 0.271 | -0.0547 | 0.538** |
|  | (-0.121 - 0.662) | (-0.633 - 0.524) | (0.0117 - 1.064) |
| realxorder | 0.0483 | 0.0284 | 0.0671 |
|  | (-0.0735 - 0.170) | (-0.207 - 0.264) | (-0.107 - 0.242) |
| treatmentxorder | 0.0215 | -0.0770 | 0.104 |
|  | (-0.126 - 0.169) | (-0.249 - 0.0948) | (-0.0997 - 0.308) |
| realxtreatmentxorder | -0.103 | 0.0409 | -0.223 |
|  | (-0.304 - 0.0980) | (-0.266 - 0.347) | (-0.494 - 0.0485) |
| Constant | 2.963*** | 2.807*** | 3.088*** |
|  | (2.674 - 3.252) | (2.368 - 3.246) | (2.787 - 3.389) |
|  |  |  |  |
| Observations | 47,490 | 21,030 | 26,460 |
| R-squared | 0.005 | 0.005 | 0.006 |

Robust ci in parentheses. *P* value for the interaction between discernment, treatment and headline order (realxtreatmentxorder) for the pooled sample is 0.315, for stage 1 it is 0.794 and for stage 2 it is 0.108.

*** *p* <0.01, ** *p* <0.05, * *p* <0.1.

1. This interaction remains significant when using STATA’s reghdfe package (which uses high-dimensional fixed effects): *β* = -0.204, 95%CI [-0.38, -0.030], *F*(7,47490) = 3.50, *p* = 0.024, and when only looking at stage 2 of data collection: *β* = -0.260, 95%CI [-0.48, -0.044], *F*(7,26460) = 3.44, *p* = 0.019. [↑](#footnote-ref-1)
